# Influence of *ApoE* Genotype and *Clock* T3111C Interaction with Cardiovascular Risk Factors on the Progression to Alzheimer’s Disease in Subjective Cognitive Decline and Mild Cognitive Impairment Patients

**DOI:** 10.3390/jpm10020045

**Published:** 2020-05-29

**Authors:** Valentina Bessi, Juri Balestrini, Silvia Bagnoli, Salvatore Mazzeo, Giulia Giacomucci, Sonia Padiglioni, Irene Piaceri, Marco Carraro, Camilla Ferrari, Laura Bracco, Sandro Sorbi, Benedetta Nacmias

**Affiliations:** 1 Department of Neuroscience, Psychology, Drug Research and Child Health-University of Florence–Viale Pieraccini 6, 50139 Florence, Italy; juri.balestrini@gmail.com (J.B.); silvia.bagnoli@unifi.it (S.B.); salvatore.mazzeo@unifi.it (S.M.); giuliagiacomucci.md@gmail.com (G.G.); sonia_padiglioni@libero.it (S.P.); irene.piaceri@gmail.com (I.P.); marco.carraro@unifi.it (M.C.); camilla.ferrari@unifi.it (C.F.); bracco.neuro@gmail.com (L.B.); sandro.sorbi@unifi.it (S.S.); benedetta.nacmias@unifi.it (B.N.); 2 IRCCS Fondazione Don Carlo Gnocchi, via di Scandicci 269, 50143 Florence, Italy

**Keywords:** Alzheimer’s disease, subjective cognitive decline, mild cognitive impairment, clock genes, *Clock*, *ApoE*, cardiovascular risk factors

## Abstract

Background: Some genes could interact with cardiovascular risk factors in the development of Alzheimer’s disease. We aimed to evaluate the interaction between *ApoE* ε4 status, *Clock* T3111C and *Per2* C111G polymorphisms with cardiovascular profile in Subjective Cognitive Decline (SCD) and Mild Cognitive Impairment (MCI). Methods: We included 68 patients who underwent clinical evaluation; neuropsychological assessment; *ApoE*, *Clock* and *Per2* genotyping at baseline; and neuropsychological follow-up every 12–24 months for a mean of 13 years. We considered subjects who developed AD and non-converters. Results: *Clock* T3111C was detected in 47% of cases, *Per2* C111G in 19% of cases. *ApoE* ε4 carriers presented higher risk of heart disease; *Clock* C-carriers were more frequently smokers than non C-carriers. During the follow-up, 17 patients progressed to AD. Age at baseline, *ApoE* ε 4 and dyslipidemia increased the risk of conversion to AD. *ApoE* ε4 carriers with history of dyslipidemia showed higher risk to convert to AD compared to *ApoE* ε4− groups and *ApoE* ε4+ without dyslipidemia patients. *Clock* C-carriers with history of blood hypertension had a higher risk of conversion to AD. Conclusions: *ApoE* and *Clock* T3111C seem to interact with cardiovascular risk factors in SCD and MCI patients influencing the progression to AD.

## 1. Introduction

Alzheimer’s disease (AD) is characterized by a slow but progressive trend, with a presymptomatic phase that can last from years to decades [1]. Subjective Cognitive Decline (SCD) is defined as a self-experienced persistent decline in cognitive capacity in comparison with the subject’s previously status, during which the subject has normal performance on standardized cognitive tests [2]. Mild cognitive impairment (MCI) concerns an objective cognitive impairment with minimal impact on instrumental activity of daily living [3], and it is considered an intermediate phase between normal cognition and dementia. MCI is associated with an increased risk of positive AD biomarkers and with an annual conversion rate of 5%–17% to AD [4,5]. For SCD, the annual conversion rate (ACR) to MCI is 3.6%–6.6%, while it is 1.5%–2.3% to dementia [4,6].

A growing amount of evidence has underlined the importance of cardiovascular health on the risk of developing AD [7,8,9]. It has been reported that approximately one-third of AD cases worldwide may be attributable to cardiovascular risk factors, including hypertension, obesity, diabetes, smoking, and physical inactivity [10].

In addition, it is well known that genetic aspects play a central role in development of AD [11]. Apolipoprotein E e4 carrier status (*ApoE* e4) is a well-defined genetic risk factor, and recently, current research has been focused on clock genes. *Clock* (*Circadian Locomotor Output Cycle Kaput*, chromosome 4q12) and *Per2* (*Period2*, chromosome 2q37.3) are part of the transcriptional-translational feedback loops regulating circadian rhythm [12]. Several polymorphisms of these genes have been recently studied to elucidate their role in sleep-wake cycle alterations, aging, psychiatric disturbance and neurodegeneration [13,14]. Moreover, *Clock* and *Per2* polymorphisms have been associated with overweight and glucose and lipid metabolism impairments [15,16]. Some studies have investigated the possible role of the *Clock* T3111C polymorphism on the quality of aging in very elderly patients [17], while other studies focused on influence of *Per2* C111G polymorphism on lipid metabolism in adults with metabolic syndrome [16]. On the basis of the above-mentioned initial findings about of the influence of these polymorphisms on cardiovascular profile, the aim of the present study was to define the interaction between *Clock* T3111C and *Per2* C111G, *ApoE* ε4, and cognitive function, in relation to cardiovascular risk factors, in SCD and MCI patients and in the progression to AD.

## 2. Materials and Methods

### 2.1. Participants and Clinical Assessment

As part of a longitudinal, clinical-neuropsychological-genetic survey on SCD and MCI, we included 74 consecutive spontaneous patients who self-referred to the Centre for Alzheimer’s Disease and Adult Cognitive Disorders of Careggi Hospital in Florence between April 1996 and May 2014. All participants underwent a comprehensive family and clinical history, general and neurological examination, extensive neuropsychological investigation, estimation of premorbid intelligence, as well as assessment of depression. A positive family history was defined as one or more first-degree relatives with documented cognitive decline. Sleep quality was assessed according to anamnestic data: we considered as “poor sleepers” patients who had difficulties in falling asleep or woke up early or have frequent sleep interruptions; patients that did not report sleep disturbances were classified as “good sleepers”. For this study, inclusion criteria were: (1) complaining of cognitive decline with a duration of ≥6 months; (2) normal functioning on the Activities of Daily Living and the Instrumental Activities of Daily Living scales; (3) unsatisfied criteria for dementia at baseline [18]; (4) attainment of the clinical endpoint, i.e., conversion to AD according to the NIA-AA [18] criteria during follow up, regardless of follow-up duration; (5) a follow-up time of more than 2 years from the baseline visit for those patients who did not develop AD. Exclusion criteria were: (1) history of head injury, current neurological and/or systemic disease, symptoms of psychosis, major depression, alcoholism or other substance abuse; (2) the complete data loss of patients’ follow-up; (3) progression to dementia other than AD.

From the initial sample, we excluded six patients: two patients had a follow-up shorter than 2 years; two diagnosed with psychiatric disturbance, and one with Fronto-Temporal Dementia, according to Neary criteria [19]; one patient received a diagnosis of Vascular Dementia [20]. Therefore, in the end 68 patients were included.

We divided this sample into two groups: 41 patients classified as SCD, according to the terminology proposed by the Subjective Cognitive Decline Initiative (SCD-I) Working Group [2] (i.e., presence of a self-experienced persistent decline in cognitive capacities with normal performance on standardized cognitive tests); 27 patients classified as MCI according to (NIA-AA) criteria for the diagnosis of MCI [3] (i.e., evidence of lower performance in one or more cognitive domains with preserved independence of function in daily life).

All patients underwent clinical and neuropsychological follow-up every 12 or 24 months. All of them were genotyped for ApoE (Apolipoprotein E), Clock and Per2.

On the basis of progression to AD during the follow-up, patients were classified respectively into converters and non-converters. All subjects gave their informed consent for inclusion before they participated in the study. The study was conducted in accordance with the Declaration of Helsinki, and the protocol was approved by the Ethics Committee (DSM study).

### 2.2. Neuropsychological Assessment

All patients were evaluated by means of an extensive neuropsychological battery [21]. The battery consisted of global measurements [Mini-Mental State Examination (MMSE)], tasks exploring verbal and spatial short- term memory (Digit Span; Corsi Tapping Test) and verbal long-term memory [Five Words and Paired Words Acquisition (FWA, PWA); recall after 10min (FWR10, PWR10); recall after 24-h (FWR24, PWR24); Babcock Short Story Immediate and Delayed Recall (BS, BSR)]; language (Token Test; Category Fluency Task); and visuo-motor functions (Copying Drawings) [21]. Visuospatial abilities were also evaluated by Rey–Osterrieth Complex Figure copy, and visuospatial long-term memory was assessed by means of recall of Rey–Osterrieth Complex Figure test [22]; attention/executive function was explored by means of Dual Task [23], Phonemic Fluency Test [24], and Trail Making Test [25]. Everyday memory was assessed by means of Rivermead Behavioral Memory Test (RBMT) [26]. All raw test scores were adjusted for age, education and gender according to the correction factor reported in validation studies for the Italian population [21,22,23,24,25,26]. In order to estimate pre-morbid intelligence, all patients were given the TIB (“Test di Intelligenza Breve”) [27], an Italian version of the National Adult Reading Test [28]. The presence and severity of depressive symptoms was evaluated by means of the 22-item Hamilton Depression Rating Scale (HRSD) [29].

### 2.3. Apolipoprotein E ε4, Clock T3111C and Per2 C111G Genotyping

A standard automated method (QIAcube, QIAGEN) was used to isolate DNA from peripheral blood samples. ApoE genotypes were investigated by high resolution melting analysis (HRMA). Two sets of PCR primers were designed to amplify the regions encompassing rs7412 [NC_000019.9:g.45412079C>T] and rs429358 (NC_000019.9:g.45411941T>C). The samples with known ApoE genotypes, which had been validated by DNA sequencing, were used as standard references. The ApoE genotype was coded as ApoE ε4− (no ApoE ε4 alleles) and ApoE ε4+ (presence of one or two ApoE ε4 alleles).

The analyses of Clock and Per2 were performed using HRMA in order to detect the 3111T/C Clock polymorphism using primers as reported [30] and the Per2 C111G polymorphism with the following primers Forward 5′-ACAGAAAGAGTCAAATGGGTGC-3′, Reverse 5′-TGTCCACATCTTCCTGCAGT-3′ with Annealing temperature 60 °C.

### 2.4. Statistical Analysis

Patient groups were characterized using means and standard deviations (SD). We tested for the normality distribution of the data using the Kolmogorov–Smirnov test. Depending on the distribution of our data, we used t-test or non-parametric Mann–Whitney U Tests for between-groups’ comparisons. We used chi-square test to compare categorical data. We analyzed survival curves using the Kaplan–Meier estimator. Finally, we used logistic regression to analyze the role of some cardiovascular risk factors in worsening cognition. All statistical analyses were performed with SPSS software v.25 (SPSS Inc., Chicago, IL, USA). The significance level was set at *p* < 0.05.

## 3. Results

### 3.1. Participants and Clinical Assessment

In the whole cohort, 32 of 68 patients (47%) were *Clock* C carriers (29 TC, 3 CC), while 13 of 68 (19%) were *Per2* G carriers (13 CG, 0 GG); 7 of 68 (10%) carried both *Clock* C and *Per2* G alleles. The genotypic distributions of the *Clock* and *Per2* genes in this sample were in Hardy–Weinberg equilibrium (*Clock* T3111C χ^2^ = 0.91, p > 0.05; *Per2* C111G χ^2^ = 0.77, *p* > 0.05). The prevalence of *Clock* T3111C and *Per2* C111G polymorphisms did not significantly differ between SCD and MCI (*Clock* T3111C: 51.2% in SCD and 40.7% in MCI; *Per2* C111G: 19.5% in SCD and 18.5% in MCI); moreover, there were not any differences in the prevalence of both *Clock* C and *Per2* G carriers in SCD and MCI groups. (Table 1).

There were no differences between *Clock* C carriers and non C carriers with regards to age at onset of symptoms, age at baseline visit, disease duration, sex, family history of AD, years of education, TIB, MMSE, and *ApoE* ε4 allele status. With respect to CV risk factors, there was a higher proportion of smokers in *Clock* C carriers than in non C carriers (19.40% vs. 2.80%, χ^2^ = 4,892, *p* = 0.027) (Table 2).

Comparing *Per2* G carriers and non G carriers, there were no differences in age at onset of symptoms, age at baseline visit, disease duration, sex, family, MMSE, and *ApoE* ε4 allele status. *Per2* G carriers had lower premorbid intelligence score on TIB (104.29 ± 10.74 vs. 109.98 ± 8.06, p = 0.049), less years of education (7 ± 3.05 vs 10.73 ± 4.51, *p* = 0.007), and lower frequency of family history of AD (15.38% vs. 60%, χ^2^ = 8.37, *p* = 0.004) (Table 2). There were no differences in CV risk factors proportion between G and non G carriers.

We did not find any differences in sleep quality between *Clock* C carriers and *Clock* non C carriers (χ^2^ = 0.136, *p* = 0.454), neither between *Per2* G carriers and *Per2* non G carriers (χ^2^ = 0.879, *p* = 0.273) (Table 2).

*ApoE* ε4+ patients have a higher proportion of history of heart disease than *ApoE* ε4− (8.70% vs. 0.00%, χ^2^ = 3.944, *p* = 0.047). We did not find any statistically significant difference between *ApoE* ε4+ and ApoE ε4− as far age at onset of symptoms, age at baseline evaluation, disease duration (time from onset of symptoms and baseline evaluation), follow-up time, familiarity, sex, education, and MMSE (Table 2).

### 3.2. Description of Sample at Follow-Up

During the follow-up, 17 patients (25%, 2 SCD and 15 MCI) converted to AD (converters) while 51 patients did not progress to AD (non-converters). Mean conversion time was 4.73 ± 3.91 years (range: 1.41–14.01, IQR = 3.71 years). Mean follow-up time of non-converters was 13.03 ± 4.48 years (range: 4.06–23.74, IQR = 6.73 years). There were no differences between converters and non-converters with respect to disease duration, sex, family history of AD, years of education, and MMSE at baseline. Converters had higher age at the onset of symptoms (68.20 ± 7.49 vs. 61.25 ± 6.21, *p* = 0.016), age at baseline (71.60 ± 6.19 vs. 63.42 ± 6.96, *p* = 0.003) and greater proportion of ApoE ε4 (56.25% vs. 25.49%, χ^2^ = 5.22, *p* = 0.022). (Table 3).

There were no significant differences between converters and non-converters in the prevalence of Clock T3111C (35.3% vs. 51.0%, χ^2^ = 1.26, *p* = 0.262) and Per2 C111G (23.5% vs. 17.6%, χ^2^ = 0.285 *p* = 0.593) polymorphism (Table 3).

In order to ascertain the effects of cardiovascular risk factors on the conversion to AD, we performed a proportional hazards regression analysis considering conversion time as time and “conversion to AD” as dependent variable. We considered as covariates age at onset, age at baseline, ApoE, Clock and Per2 genotype, hypertension, diabetes, dyslipidemia, heart disease, and smoking habit. Dyslipidemia (*p* = 0.041, HR = 3.08, 95% I.C. = 1.05: 9.09), age at baseline (*p* = 0.001, HR = 1.16, 95% I.C. = 1.07:1.27) and ApoE ε4 (*p* = 0.001, HR = 6.21, 95% I.C. = 2.04:18.9) were statistically significantly associated with an increased likelihood of conversion to AD (Table 4).

### 3.3. Relationship between ApoE and Dyslipidemia

**Figure 1:**
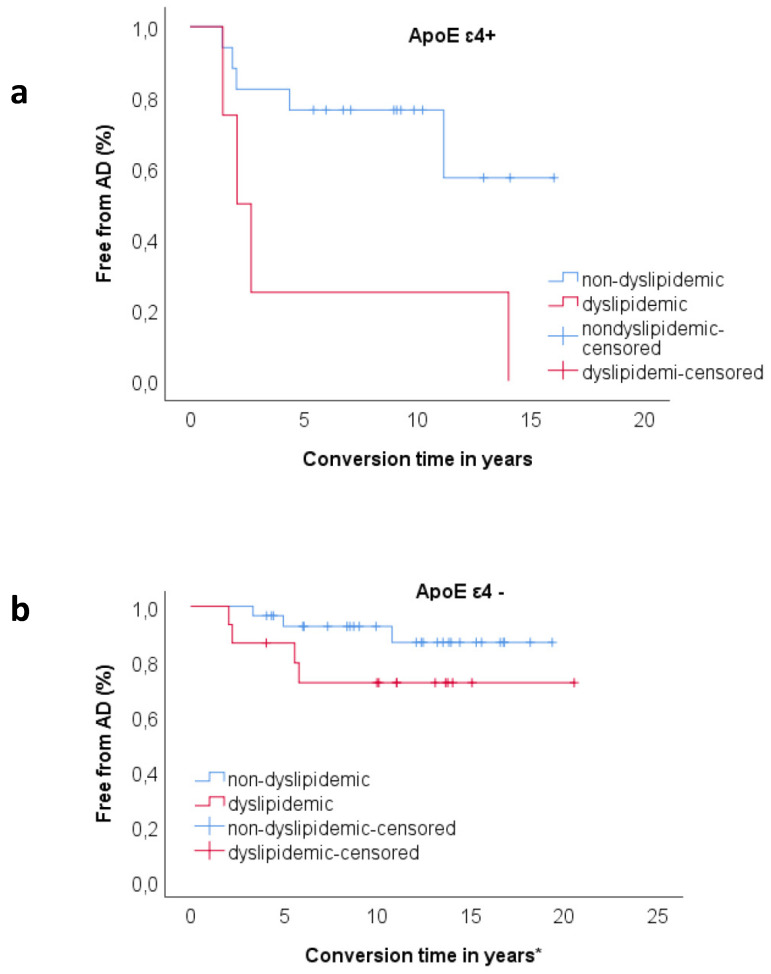
(**a**) Kaplan–Meier survival analysis for comparisons of proportion of progression to AD between dyslipidemic (n = 4) and non-dyslipidemic (n = 17) patients in *ApoE ε*4+ carrier group. Proportion of progression was higher in dyslipidemic group (100.00%) compared to non-dyslipidemic (29.40%). The pairwise log rank comparisons showed significant difference in survival distributions for the dyslipidemic vs. non-dyslipidemic (χ^2^ = 4.42, *p* = 0.036). (**b**) Kaplan–Meier survival analysis for comparisons of proportion of progression to AD between dyslipidemic (n = 15) and non-dyslipidemic (n = 29) patients in *ApoE ε4*− carrier group. The pairwise log rank comparisons showed no significant difference in survival distributions for the dyslipidemic vs. non-dyslipidemic (χ^2^ = 1.92, *p* = 0.166). * For censored cases (non-converters), conversion time indicates follow-up time.

In order to explore the relationship between dyslipidemia and ApoE genotype, we divided the sample according to ApoE ε4 status (ε4+ and ε4−). In the ε4+ sample, a Kaplan–Meier survival analysis showed significant difference in survival distributions between patients with history of dyslipidemia and patients without history of dyslipidemia (χ^2^ = 4.42, *p* = 0.036), as 100% of dyslipidemic patients and 29.4% of non-dyslipidemic patients converted to AD (Figure 1a). When we performed the same analysis on the ε4− sample, we found no statistically significant effect of dyslipidemia on rate of progression to AD (χ^2^ = 1.92, *p* = 0.166) (Figure 1b).

**Figure 2:**
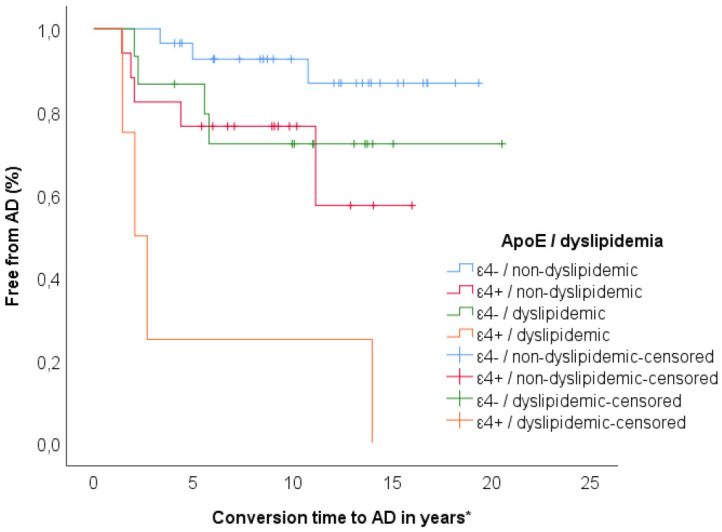
Kaplan–Meier survival analysis for comparisons of patients ranked according to history of dyslipidemia and ApoE genotype (non-dylipidemic/ε4−, n = 29; dyslipidemic/ε4−, n = 17; non-dylipidemic/high/ε4+, n = 15; dylipidemic/ε4+, n = 4). Proportion of progression was higher in dylipidemic/ε4+ (100.00%) compared to non-dylipidemic/ε4− (10.30%, χ^2^ = 25.47, *p* < 0.001), non-dylipidemic/ε4+ (29.40%, χ^2^ = 4.42, *p* = 0.036), and dylipidemic/ε4− (26.7%, χ^2^ = 7.64, *p* = 0.006). Proportion of progression in non-dylipidemic/ε4+ group (26.7%) was higher than non-dylipidemic/ε4− (10.3%, χ^2^ = 3.73, *p* = 0.05). * For censored cases (non converters) conversion time indicates follow-up time.

Finally, we ranked the whole sample according to history of dyslipidemia and ApoE genotype (non-dyslipidemic/ε4−, n = 29; non-dyslipidemic/ε4+, n = 17; dyslipidemic/ε4−, n = 15; dyslipidemic/ε4, n = 4) and Kaplan–Meier survival analysis was conducted to compare the proportions of conversions in the four different groups. Patients in dyslipidemic/ε4+ group had a higher rate of conversion to AD compared to non-dyslipidemic/ ε4− (χ^2^ = 25.47, *p* < 0.001), non-dyslipidemic/ε4+ (χ^2^ = 4.42, *p* = 0.036) and dyslipidemic/ε4− (χ^2^ = 7.64, *p* = 0.006). Proportion of conversion in non-dyslipidemic/ε4+ was higher as compared to non-dyslipidemic/ε4− (χ^2^ = 3.73, *p* = 0.05). There was no significant difference between non-dyslipidemic/ε4−, dyslipidemic/ε4− and dyslipidemic/ε4− (Figure 2).

### 3.4. Relationship between Clock and Per2 and Risk Factors on Progression to AD

In order to explore the relationship between CV risk factors and Clock polymorphism, we divided the sample according to Clock genotype status (C carriers and non C carriers). For each sample, we performed a proportional hazards regression analysis considering conversion time as time; “conversion to AD” as dependent variable; and age at onset, age at baseline, ApoE genotype, hypertension, diabetes, dyslipidemia, heart disease, and smoking habit as covariates. In the C carriers sample, hypertension at baseline (*p* = 0.025, HR = 3.625) was statistically significantly associated with an increased likelihood of conversion to AD (Table 4). In the Clock non C carriers, none of the CV factors were included in the model as only age at baseline was statistically significantly associated with high risk of progression to AD.

**Figure 3:**
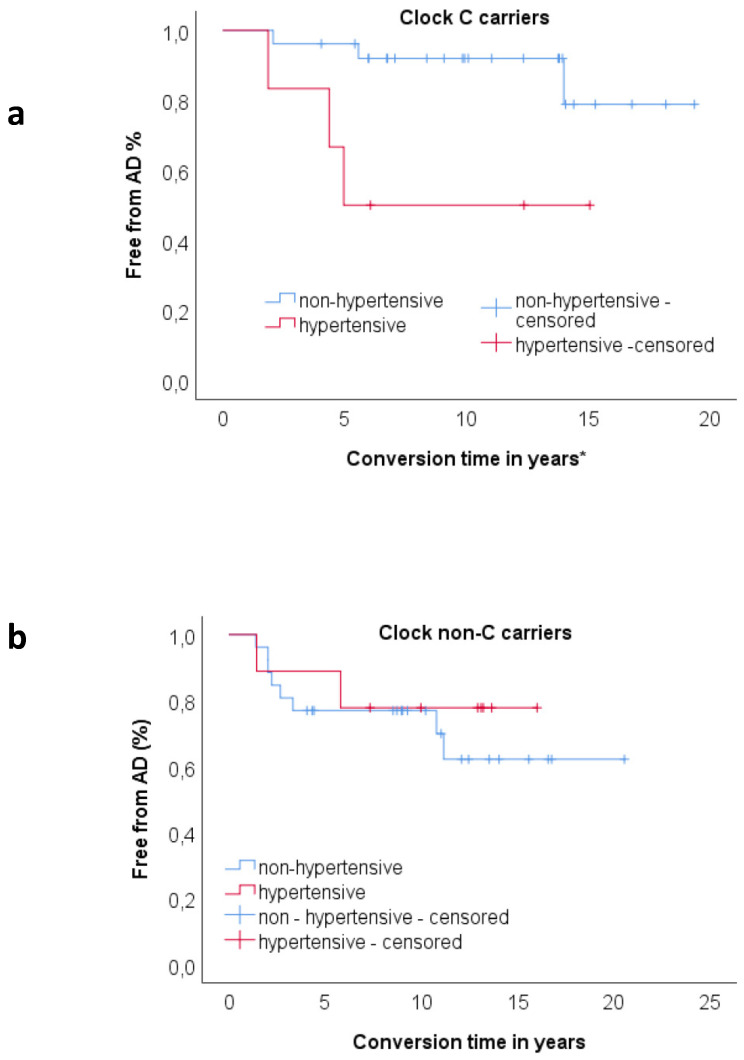
(**a**) Kaplan–Meier survival analysis for comparisons of proportion of progression to AD between hypertensive (n = 6) and non-hypertensive (n = 26) patients in *Clock* C carriers group. Proportion of progression was higher in hypertensive group (50.00%) compared to non-hypertensive (11.50%). The pairwise log rank comparisons showed significant difference in survival distributions for the hypertensive vs. non-hypertensive (χ^2^ = 5.77, *p* = 0.017). (**b**) Kaplan–Meier survival analysis for comparisons of proportion of progression to AD between hypertensive (n = 9) and non-hypertensive (n = 26) patients in *Clock* non C carriers group. The pairwise log rank comparisons showed no significant difference in survival distributions for the hypertensive vs. non-hypertensive (χ^2^ = 0.323, *p* = 0.570). * For censored cases (non converters) conversion time indicates follow-up time.

A Kaplan–Meier survival analysis showed significant difference in survival distributions between patients with history of hypertension and patients without history of hypertension (χ^2^ = 4.42, *p* = 0.036) only in the Clock C carriers sample (Figure 3a). In this group, 50% of hypertensive patients and 11.5% of non-hypertensive patients converted to AD. When we performed the same analysis on the Clock non C carriers sample, we found no statistically significant effect of hypertension on rate of progression to AD; (Figure 3b).

We performed the proportional hazard regression analysis ranking patients according to Per2 polymorphism, including as covariates age at onset, age at baseline, ApoE genotype, hypertension, diabetes, dyslipidemia, heart disease, and smoking. In the G carriers sample, none of the covariates showed a statistically significant effect on risk of conversion to AD. In the Per2 non G carriers, none of the CV factors were included in the model as only age at baseline was statistically significantly associated with high risk of progression to AD (*p* = 0.010, HR = 1.185).

## 4. Discussion

This study investigated the interaction between genetic features (*Clock* T3111C, *Per2* C111G polymorphisms, and *ApoE* genotype) and cardiovascular risk factors in a sample of SCD and MCI patients. We are not aware of any previous studies exploring this topic on these groups of patients.

The frequency of these polymorphisms in our cohort was similar to prevalence data reported in a previous study on healthy Italian population [31].

We found that *Clock* T3111C carriers were more frequent smokers compared to non-carriers of the polymorphism. Our result may suggest an implication also of Clock T3111C on nicotine dependence. Other researches have shown that clock genes are associated with substance abuse, including alcohol, cocaine and cannabis [32]. In fact, circadian genes have a direct role in in the regulation of dopaminergic transmission, especially in reward circuitry. This evidence could represent the biological substrate for the role of Clock genes in the development of addicted behaviours [33,34].

We found a correlation between *Per2* C111G polymorphism with years of education and family history of AD. This result confirms previous evidence by our group [35], but it is difficult to interpret this finding due to the absence of previous works on this issue.

For future researches on wider sample, we will aim to clarify our current findings. With regard to cardiovascular risk factors, we did not find any association with *Per2* C111G.

We found that *ApoE* ε4 carriers had more frequent history of heart disease than ε4 non carriers. Our result is supported by previous studies. In particular, two different meta-analysis [36,37] showed a different distribution of coronary disease risk according to *ApoE* genotype. Furthermore, this association could be independent from other cardiovascular risk factors, as we did not find any correlation with diabetes, dyslipidemia, smoking, or hypertension.

A multivariate analysis showed that age at baseline, *ApoE* e4 and dyslipidemia increase the risk of progression to AD.

With regard to age, this is not surprising as it is recognized to be the major risk factor for AD [38,39].

The effect of *ApoE* ε4 allele on risk of progression to AD has been widely demonstrated by previous studies [40,41,42,43].

Last, lipid disorders have been said to have a role in cognitive impairment and their treatment has been studied as a prevention tool, but evidence about this topic is not yet conclusive [44].

The interaction between *ApoE* ε4 and dyslipidemia on cognition is not yet completely understood [36]. In order to explore this point, we ranked the patients according to *ApoE* genotype. The effect of dyslipidemia on progression to AD was confirmed only in *ApoE* ε4 carriers. Furthermore, we showed that patients who were *ApoE* ε4 carriers and had history of dyslipidemia showed higher risk to convert to AD both compared to *ApoE*ε4− groups and *ApoE* ε4+ without dyslipidemia patients. According to this analysis, dyslipidemia could be synergistic with E4 carrier status in contributing to AD pathogenesis as reported by other authors [44].

Previous studies suggested an association between *Clock* gene polymorphisms with different cardiovascular risk factors, as well as with the cognitive state [15,16,17]. In order to investigate a possible interaction between *Clock* T3111C and cardiovascular risk factors, we ranked patients according to *Clock* genotype. We found that *Clock* C carriers with history of blood hypertension had a higher risk of conversion to AD than *Clock* C carriers without hypertension. This difference was not detected in the *Clock* non C carrier group. No other work, to the best of our knowledge, has previously studied the possible influence of *Clock* gene polymorphisms on the effect of hypertension in conversion to AD in this particular group of patients.

A limitation of this study is the small size of our cohort. In future we aim to expand our sample, also including a healthy control group, to support our present results.

Secondly, the lack of quantitative data about dyslipidemia and hypertension did not allow us to understand if our results might be different according to the level of blood lipids and blood pressure. Another limitation of this study is that AD diagnosis was not supported by AD biomarkers. Future researches including cerebrospinal fluid amyloid beta, tau and p-tau levels or neuroimaging data, as amyloid PET, could provide interesting and additional information. Finally, as it is a single-center study, there may be biases with regard to assessment and diagnosis procedures. On the other hand, this study has some remarkable strengths. First of all, to the best of our knowledge, this is the first prospective study that assessed the interaction of these clock genes polymorphisms with cardiovascular factors on the risk or progression to AD in cohort of well-defined SCD and MCI patients. The second strength is the very long mean follow-up time of 13 years. Moreover, non-converter patients had a longer follow-up time than patients who converted to AD. This is crucial information as follow-up time could influence rate of conversion to AD. The long follow-up time in our sample allows us to minimize the risk of classifying as stable subjects carrying an Alzheimer pathology who will convert later. Indeed, the present study suggests an association between *ApoE* genotype and *Clock* T3111C with different cardiovascular risk factors in a cohort of SCD and MCI patients. This interaction could influence the progression to AD in this group of patients. Understanding the mechanisms by which genetic and cardiovascular risk factors contribute to AD could inspire the development of new personalized therapeutic approaches for this disease.

## Figures and Tables

**Table 1:** Comparison between prevalence of *Clock* and *Per2* polymorphisms in SCD and MCI individuals.

Features	SCD	MCI	*p*
*Per2* G carriers–units (%)	8 (19.5%)	5 (18.5%)	0.919
*Clock* C carriers-units (%)	21 (51.2%)	11 (40.7%)	0.397
*Per2* G carriers and *Clock* C carriers-units (%)	4 (9.75%)	3 (8.10%)	0.843

*p* indicates level of significance for comparison between groups (statistical significance at *p* < 0.05, in bold characters).

**Table 2:** Demographic data according to *Per2*, *Clock*, *ApoE*4.

Features	*Per2*			*Clock*			*ApoE*		
	non G (n = 55)	G (n = 13)	*p*	non C (n = 36)	C (n = 32)	*p*	ε4− (n = 45)	ε4+ (n = 23)	*p*
**SCD (% within SCD)**	80.5%	19.5%	-	48.8%	51.2%	-	68.3%	31.7%	-
**MCI (% within MCI)**	81.5%	18.5%	-	59.3%	40.7%	-	63%	37%	-
**Conversion to AD (SCD) (% in consideration of carrier status)**	0.0%	25.0%	-	5.0%	4.8%	-	3.6%	7.7%	-
**Conversion to AD (MCI) (% in consideration of carrier status)**	59.1%	40.0%	-	62.5%	45.5%	-	35.3%	90%	-
**Age at baseline (±SD) in years**	64.03 ± 9.12	65.22 ± 7.26	0.739	64.29 ± 9.33	63.69 ± 8.60	0.777	63.38 ± 9.08	65.38 ± 8.75	0.354
**Age at onset (±SD) in years**	59.69 ± 10.02	62.38 ± 7.96	0.562	60.56 ± 10.87	59.25 ± 8.64	0.694	59.38 ± 10.12	61.83 ± 9.17	0.228
**Follow-up time (±SD) in years**	10.87 ± 4.23	12.60 ± 4.68	0.234	11.34 ± 4.31	10.90 ± 4.45	0.815	13.00 ± 4.63	10.54 ± 4.71	0.065
**Disease duration (±SD) in years**	4.36 ± 3.63	2.83 ± 2.33	0.090	3.73 ± 3.65	4.44 ± 3.18	0.195	4.00 ± 4.85	3.55 ± 2.00	0.800
**Sex (females, males)**	37. 17	10. 3	0.552	26. 10	22. 10	0.754	33, 12	15, 8	0.487
**Family history of AD (%)**	61.11%	15.38%	0.003 *	41.66%	62.5%	0.086	43.18%	65.22%	0.105
**Education in years (±SD)**	10.72 ± 4.55	7 ± 3.05	0.007 *	9.86 ± 4.80	10.19 ± 4.21	0.737	9.73 ± 4.51	10.57 ± 4.54	0.455
**MMSE (±SD)**	28.46 ± 1.66	28.31 ± 1.49	0.583	28.09 ± 1.78	28.81 ± 1.33	0.064	28.34 ± 1.78	28.595 ± 1.26	0.839
***ApoE* ε4 (%)**	35.18%	23.07%	0.404	30.55%	37.5%	0.546	-	-	-
Diabetes (%) *	5.60%	7.70%	0.770	8.30%	3.10%	0.362	2.20%	13.00%	0.730
Hypertension (%) *	22.20%	23.10%	0.947	25.00%	18.8%	0.535	22.20%	21.70%	0.964
Dyslipidemia (%) *	30.80%%	23.10%	0.585	30.60%	26.70%	0.728	34.10%	18.20%	0.178
Heart Disease (%) *	3.80%	0.00%	0.477	2.90%	3.10%	0.949	0.00%	8.70%	**0.047**
Smoking (%) *	11.30%	7.70%	0.703	2.80%	19.40%	**0.027**	6.80%	17.40%	0.179

Values quoted in the table are mean (±SD) or (%) or units. *p* indicates level of significance for comparison between groups (statistical significance at *p* < 0.05, in bold characters). * every risk factor was evaluated at the baseline for statistical purpose.

**Table 3:** Comparison of demographic and clinical data between converters and non-converters.

Features	Converters (n = 17)	Non Converters (n = 51)	*p*
**Prevalence *Per2* G (%)**	23.5%	17.6%	0.593
**Prevalence *Clock* C (%)**	35.3%	51.0%	0.262
**Age at baseline (±SD) in years**	70.7 (± 6.3)	61.8 (±8.6)	**<0.01**
Age at onset * (±SD) in years	67.4 (± 7.5)	57.9 (±9.4)	**<0.01**
**Follow-up time (±SD) in years**	9.6 (± 4.7)	13.03 (±4.5)	**<0.01**
**Disease duration (±SD) in years**	3.3 (± 3.6)	4.0 (±4.3)	0.602
**Sex (females, males)**	12,5	36,15	1.000
**Family history of AD (%)**	52.9%	51.0%	0.889
**Education in years (±SD)**	8.4 (±3.9)	10.6 (±4.6)	0.059
**MMSE (±SD)**	28.1 (±1.1)	28.5 (±1.8)	0.399
***ApoE* ε4 (%)**	58.8%	25.5%	**0.012**
**Hypertension (%)**	29.4%	19.6%	0.399
**Diabetes (%)**	11.8%	3.9%	0.234
**Dyslipidemia (%)**	47.1%	22.4%	0.053
**Heart disease (%)**	11.8%	0.0%	**0.014**
**Smoking (current) (%)**	0.0%	14.0%	0.103
**Chronic kidney disease (%)**	0.0%	0.0%	-

Values quoted in the table are mean (± SD) or (%). *p* indicates level of significance for comparison between groups (statistical significance at *p* < 0.05, in bold characters). * onset of memory problems (not overt dementia).

**Table 4:** Proportional hazards regression analysis.

	B	*p*	HR	95% C.I.	
				Lower	Upper
**Whole sample**					
*ApoE* e4	1.826	0.001	6.212	2.045	18.872
Age at baseline	0.151	0.001	1.163	1.067	1.269
Dyslipidemia	0.126	0.041	3.083	1.045	9.099
***Clock* C carriers**					
Hypertension	3.265	0.025	26.18	1.510	454.039
***Clock* non C carriers**					
Age at baseline	0.204	0.002	1.23	1.078	1.394
*ApoE*	2.111	0.009	8.25	1.712	39.791

Regression Coefficients (B), *p*-value (*p*), Hazard Ratio (OR) and 95% Confidence Intervals (95% CI) for covariates included in the proportional hazards regression model are reported (significant differences at *p* < 0.05).
